# The Correlation of Swedish Snus, Nicotine Pouches and Other Tobacco Products with Oral Mucosal Health and Salivary Biomarkers

**DOI:** 10.3390/dj10080154

**Published:** 2022-08-17

**Authors:** Sintija Miluna, Ricards Melderis, Loreta Briuka, Ingus Skadins, Renars Broks, Juta Kroica, Dagnija Rostoka

**Affiliations:** 1 Department of Prosthetic Dentistry, Riga Stradins University, LV-1007 Riga, Latvia; 2 Department of Biology and Microbiology, Riga Stradins University, LV-1007 Riga, Latvia; 3 Emergency Department, Pauls Stradins Clinical University Hospital, LV-1002 Riga, Latvia; 4 Department of Medicine, Riga Stradins University, LV-1007 Riga, Latvia

**Keywords:** oral health prevention, smokeless tobacco, e-cigarettes, oral mucosa, oral health, white lesions

## Abstract

In society, tobacco products, such as e-cigarettes, and smokeless tobacco products, such as snus and nicotine pouches, are becoming more attractive. There is still a lack of information regarding the effects of these products on the oral mucosa and oral saliva biomarkers. The aim of this study is to evaluate oral mucosa and the presence of inflammatory biomarkers IL-6, IL-1, IL-8, TNF alpha and LRG-1 in saliva. Respondents were divided in four groups based on their tobacco product usage. Oral examination was carried out, saliva samples were taken, and the detection of IL-6, IL-8, IL-1, TNF alpha and LRG-1 levels in saliva was carried out. Out of the tobacco users, 30.8% were snus users, 48.7% were cigarette users and 20.5% were e-cigarette users. The control group was composed of respondents who did not use any tobacco products. E-cigarettes were used more by women, but snus was used more by men. Mucosal changes were seen in the group of snus users, and mucosal changes were only seen in men who had used 5–10 tobacco units per day for 5–10 years. Increased IL-6 levels in saliva were detected in respondents who also experienced mucosal changes. Mucosal changes were white, leathery and localized at the site where snus sachets were placed. Saliva, as an easily available biofluid, could be used as a first tool to detect potentially precancerous signs, but the LRG1 marker cannot be used as a prognostic marker.

## 1. Introduction

The use of tobacco products kills around 8 million people a year; nevertheless, new forms of tobacco products are being created that are more legally accessible for young adults and children under the age of 18 [1]. In Latvia, the rate of daily tobacco consumption is 30%, and this is one of the highest rates in the European Union [2]. In Europe, the tobacco use prevalence is over 29%, which is the highest value reported by the World Health Organization [3]. New forms of tobacco products, such as smokeless tobacco (Swedish snus), are highly popular among young adults. In society, there is an unfounded myth that this type of tobacco is harmless and healthier. These new tobacco products, considered to be “harmless products”, will slowly cause oral manifestations in individuals, such as oral cancer. Moreover, the COVID-19 pandemic has had a significant negative impact on people’s lifestyles [4]. It was found that, during quarantine, people ate more carbohydrates and their alcohol and tobacco consumption was higher [5].

In Latvia, the sale of Swedish snus is prohibited, but many hockey and floorball players use this product daily. There are a number of reasons why athletes use smokeless tobacco products, for example, it provides satisfaction and a psychological reward to athletes [6], and nicotine may enhance their performance and has a stimulatory effect as a legal dopant because of the release of catecholamines [7]. The use of nicotine as a doping agent and stimulant was investigated by the World Anti-Doping Agency in 2021 [8].

In 2019, nicotine pouches were sold in Latvian markets for the first time, and once they became legal, a lot of smokeless tobacco users switched to nicotine pouches [9]. Mass-market manufacturers who sell these products play a big role in advertising this product as a tobacco-free product that is easy to use and healthier than cigarettes. This method of advertisement has been shown to influence young people effectively, as they have started to use this product as it has become trendier [10].

Swedish snus sachets and nicotine pouches are placed under the upper or lower lip. In the regions of tobacco placement, white mucosal lesions appear—parakeratosis, leukoedema, cutaneous lichen simplex chronicus or leukoplakia-squamous cell hyperplasia [11]. Other oral manifestations include periodontitis, gingivitis, wound-healing problems, pyogenic granuloma, the discoloration of teeth and enamel abrasion [12]. The WHO Framework Convention on Tobacco Control recommends monitoring the effects of smokeless tobacco on health and other aspects, because smokeless tobacco products are used worldwide by more than 356 million people, and the marketing of these products targets young people under the age of 18 [13].

Some biomarkers, for example, IL-6, could be used in oral health screening, especially for early stage oral precancer lesions [14]. Pro-inflammatory cytokine (IL-1, IL-6, IL-8 and TNF- Alpha) and anti-inflammatory cytokine (IL-4, IL-10 and IL-13) levels are higher in individuals with oral cancer [15,16]. There is evidence that using IL-6 as a marker could be an indicator for periodontitis, leukoplakia and tobacco consumption habits [17,18]. In their recent study, Chang et al. found that LRG1 levels were significantly altered in the plasma of patients with oral cancer [19]. More biomarkers could be used as valuable early warning signs for users and to help dental practitioners to provide patients with information and to help to cease tobacco usage. Early oral changes in tobacco users could be used to provide early treatment and prophylaxis.

The aim of the study is to perform oral examinations in different tobacco users and to detect and compare IL-6, IL-1, IL-8, TNF alpha and LRG-1 levels in the saliva of different tobacco users and of participants who did not use tobacco or tobacco products.

## 2. Materials and Methods

### 2.1. Research Ethics

This cohort study was approved by the Ethics Committee of Rīga Stradiņš University No. 22/28.01.2016, and all of the participants signed informed consent regarding the collection of biological material and the photographic publicity of their oral cavity.

### 2.2. Data Collection and Research Design

An online retrospective survey (added as a Supplementary Material) regarding dental health, dietary habits and tobacco consumption habits was made and published in a social network group (Facebook) via a university student council group from August 2020 to September 2021 using a self-administered questionnaire. Participation in the questionnaire (study) was voluntary. The questionnaire was divided into 5 segments: the first segment regarded questions about sex, age and occupation. The second segment involved questions about dental health (how often do you brush your teeth, what kind of dental materials do you use, do you floss, how regularly you go to the dentist, etc.). The third segment involved questions about diet (how many sugary drinks (mL) do you consume per day, how many snacks (g) do you consume per day, how much alcohol (glasses) are drunk per week, are you vegetarian, etc.). The fourth segment involved questions about health and systemic diseases (do you take any medicine that needs to be taken daily, do you have asthma, diabetes, GERD, heart disease, are you pregnant, etc.). The fifth segment involved questions about tobacco consumption (do you use tobacco or tobacco products, what kind of product you use, how many tobacco units per day do you consume (1 cigarette/1 pouch/1 sachet is 1 unit), for how many years have you used this product, where do you place this product (snus, nicotine pouches), etc.

Respondents who were 18–35 years old, did not have any systemic diseases or medical conditions, did not take any daily medicine, were not pregnant and had not used antibiotics for at least 6 months were invited to take part in the research and to undergo an oral examination (one examinator/dentist) in a dental practice in Riga, Latvia.

Participants were divided into 4 groups according to their consumption of tobacco products, which was based on their answers from the previous questionnaire: the smokeless tobacco group, the cigarettes group, the e-cigarettes group and non-tobacco users. Only respondents who had used tobacco products for more than 2 years were included in the study. In the ST group, participants that used smokeless tobacco (snus) and/or nicotine pouches were included. In the NT group, participants that did not smoke or use any kind of tobacco products daily or occasionally were included.

### 2.3. Oral Examination

Overall, 76 respondents underwent oral mucosal examination. Clinical charts regarding caries and fillings were made. The gingival health index was determined. Oral mucosa (tongue, cheeks, lips, palate and gingiva) was inspected perceptibly, and unusual lesions were photographed (Canon, EOS 80D, Oita, Japan). Patients who had periodontitis were excluded from the study.

### 2.4. Measured Variables

#### 2.4.1. Inflammatory Biomarkers (IL-1, IL-6, IL-8 and TNF Alpha) Detection

A saliva sample (5 mL) was collected from each respondent and placed in an Eppendorf tube (5 mL, Sigma-Aldrich, Hamburg, Germany). Samples were centrifuged for 5 min with 10,000 rpm (Hermle, Z446K, Gosheim, Germany) to remove cellular debris. The supernatant was transferred to a fresh Eppendorf tube and frozen for −80 °C until all of the samples were collected from the participants. Concentrations of IL-1 beta, IL-6, IL-8/CXCL8 and Tumor Necrosis Factor alpha were determined using ELISA kits (Sigma-Aldrich, Gosheim, Germany). All ELISA kits worked by the same principle. Saliva samples and standards were added to the ELISA plate and incubated at room temperature for 2.5 h with gentle shaking. After incubation, each well was washed 4 times with an assay wash buffer and decanted. Detection antibodies were added to each well and incubated in previous conditions for 1 h. The washing steps were repeated, and streptavidin solution was added and incubated for 45 min. After the 3rd washing step, substrate reagents were added and incubated in the dark for 30 min followed by the addition of a stop solution. Absorbance was read at 450 nm using a Tecan Infinite F50 absorbance microplate reader (Tecan, Zurich, Switzerland). The results were calculated using a Four Parameter Logistic Curve based on kit-provided standards.

#### 2.4.2. LRG1 Detection

Saliva samples and standards were added to the ELISA plate and incubated at room temperature for 2 h. After incubation, each well was washed 6 times with an assay wash buffer and decanted. Biotinylated Human LRG-1 antibodies were added to each well and incubated in previous conditions for 1 h. The washing steps were repeated, and the streptavidin–peroxidase conjugate was added and incubated for 30 min. After the 3rd washing step, chromogen substrate reagents were added and incubated in the dark for 20 min, followed by the addition of a stop solution. Absorbance was read at 450 nm with 570 nm wavelength correction using a Tecan Infinite F50 absorbance microplate reader (Switzerland). The results were calculated using a Four Parameter Logistic Curve based on kit-provided standards.

### 2.5. Statistical Analysis

Statistical analysis was carried out with IBM SPSS statistics 27 (IBM Corp, Chicago, IL, USA). For non-parametric tests, the Mann–Whitney U test was used. For correlations, Spearman and Pearson correlation was used. Phi and Cramer’s coefficients were detected. Additionally, 95% confidence intervals were used and associated with the *p* = value; the significance level was 5%.

## 3. Results

### 3.1. Results of Tobacco and Control Groups

In total, 152 respondents completed the survey, but based on inclusion criteria, 76 respondents were included in the further study; 58% were women (*n* = 88) and 42% were men (*n* = 64). Other respondents (*n* = 76) were rejected (due to being older or younger than 18–35 years old, having systemic diseases, being pregnant, using daily medicine, etc.).

Oral examination and saliva sample collection was completed for 76 respondents. The average age of the participants was 24.46 years. In total, 50% were women (*n* = 38), and 50% were men (*n* = 38). The average age for female respondents was 23.71, but for men, it was 25.21. A total of 48.7% (*n* = 37) were non-tobacco users, and of these, 59.5% were women and 40.5% were men. Of all of the respondents, 15.8% (*n* = 12) were snus users, and of these, 16.7% were women and 83.3% were men. A total of 25% (*n* = 19) were cigarette users, and of these, 47.4% were women, and 52.6% were men. Additionally, 10.5% (*n* = 8) were e-cigarette users, and of these, 62.5% were women, and 37.5% were men. Out of the tobacco users, 30.8% were snus users, 48.7% were cigarette users and 20.5% were e-cigarette users (Table 1).

Out of all of the respondents, 30.3% had used tobacco products for 2–5 years (*n* = 23), 14.5% had used tobacco products for 5–10 years (*n* = 11) and 6.6% (*n* = 5) had used tobacco products for more than 10 years. Additionally, 17.91% of all of the respondents used 10 tobacco units per day (*n* = 14), 17.69% of all of the respondents used 10–20 tobacco units per day (*n* = 6) and 5.12% used more than 20 tobacco units per day (*n* = 4).

### 3.2. Results of Mucosal Changes

It was found that age was not related to mucosal changes, and this result was not statistically significant (*p* > 0.05). There was a statistically significant association between sex and mucosal changes (*p* < 0.05), and these changes were seen in male respondents, but according to the Phi coefficient (0.285), this was a weakly positive relationship. There was a statistically significant association between tobacco product type and mucosal changes (*p* < 0.05), with a Cramer’s V coefficient of 0.417, which means that the association was strong (Table 2).

**Figure 1:**
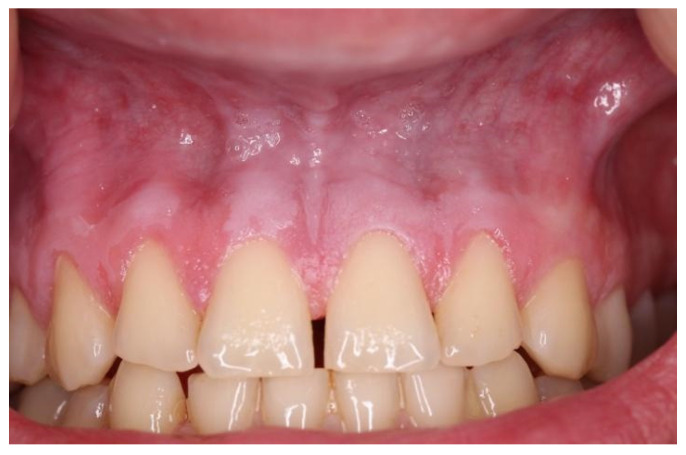
White, leathery lesion above tooth nr. 13 to tooth nr. 22.

**Figure 2:**
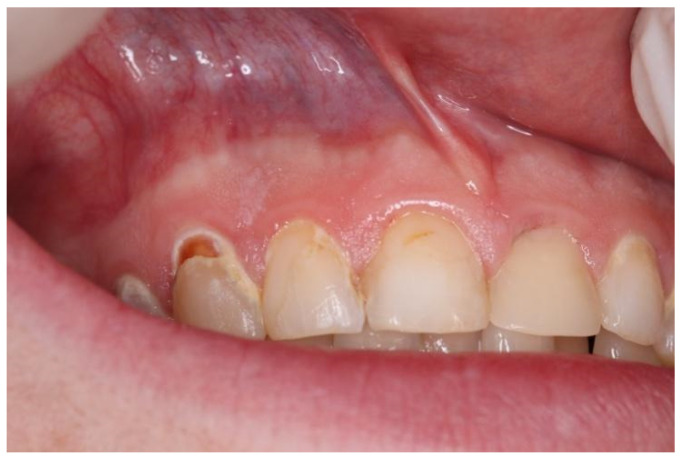
White, round lesion above tooth nr. 13 and nr. 12.

**Figure 3:**
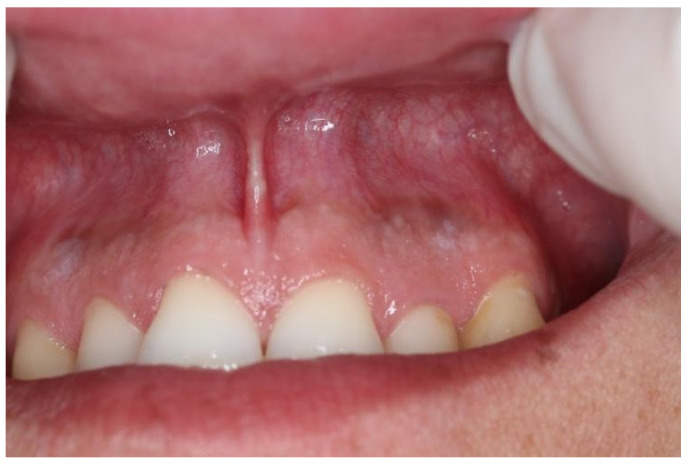
White, grainy lesion above tooth nr. 13/12 and tooth nr. 22/23.

**Figure 4:**
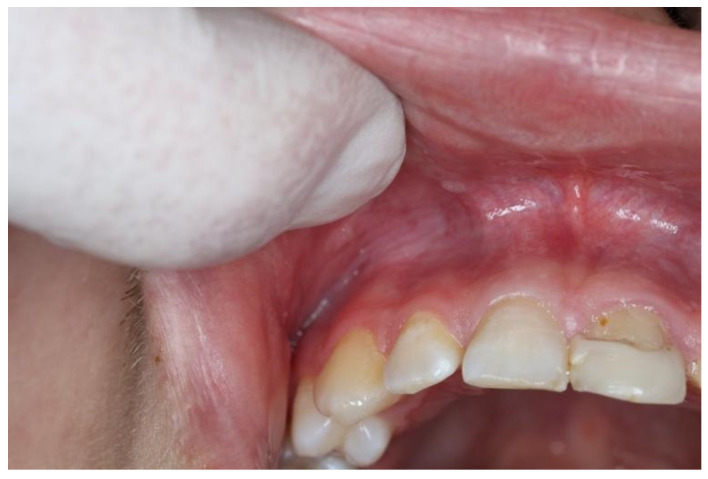
White, linear lesions in the right vestibule.

Snus group respondents displayed a tendency to experience mucosal changes. Mucosal changes were detected in nine respondents (11.8%) (Table 3). According to the respondents’ answers to the questionnaire, visual mucosal changes were seen in the places where snus or nicotine pouches were placed. In one respondent, lesions were white, localized and leathery and were present from tooth number 13 to 22 (Figure 1). Another respondent had round, white, dot-like lesions above tooth number 13 and 12 (Figure 2). Other respondents had white, round lesions above tooth number 13/12 and 22/23 (Figure 3). Respondent (Figure 4) had white, linear lesions in the sublabial vestibule. Mucosal changes were shown to have a correlation between how many years tobacco products were used, and these results were statistically significant (*p* < 0.05); the Phi coefficient was 0.41, which means that this relationship was strong. Respondents who had used tobacco products for 5–10 years had a tendency to experience mucosal changes. There was a statistically significant correlation between mucosal changes and the number of tobacco units used daily (*p* < 0.05). Respondents who used 5–10 tobacco units per day had a tendency to experience mucosal changes.

### 3.3. Results of Inflammatory Biomarkers and LRG1

The average IL-1 levels were 50.42 pg/mL, IL-6: 76.77 pg/mL, IL-8: 403.77 pg/mL and TNF alpha: 130.47 pg/mL. The average LRG1 levels were 284.93 mg/mL. The mean values in the tobacco product group and non-tobacco group differed. The highest values for IL-1 were in the snus group (105.14 pg/mL); non-tobacco users had the lowest results (21.32 pg/mL); in the e-cigarette group, the value was 78.71 pg/mL; and in the cigarette group, 49.69 pg/mL. The highest values for IL-6 were in the snus group (177.43 pg/mL), then the e-cigarette group (107.39 pg/mL), the cigarette group (59.00 pg/mL) and the non-tobacco group (40.54 pg/mL). The highest values for IL-8 were in the snus group (767.43 pg/mL), then the e-cigarette group (445.09 pg/mL), the cigarette group (355.51 pg/mL), and for the non-tobacco users, the results were 281.85 pg/mL. The highest values for TNF-alpha were in the snus group (445.08 pg/mL), then the cigarette group (66.59 pg/mL), the e-cigarette group (66.60 pg/mL) and the non-tobacco group (60.22 pg/mL). The highest values for LRG1 were comparable in all of the tobacco groups: snus group: 306.35 mg/mL, cigarette group: 300.76 mg/mL and e-cigarette group: 285.78 mg/mL. For non-tobacco users, the value was 265.53 mg/mL (Table 4).

IL-1, IL-8, TNF alpha and LRG1 and their correlations with mucosal changes were not statistically significant (*p* > 0.05), but IL-6 levels had a statistically significant correlation with oral mucosal changes (*p* < 0.05). The tobacco product type had no statistically significant association with increasing levels of IL-1, IL-8, TNF alpha and LRG-1 (*p* > 0.05), but there was a statistically significant correlation between increasing IL-6 levels and tobacco product usage (*p* < 0.05). There was no statistically significant correlation between IL-1, IL-6, IL8, TNF alpha and LRG-1 levels and how many years tobacco products were used (*p* > 0.05) or how many tobacco units were used per day (*p* > 0.05) (Table 5).

## 4. Discussion

In the present cohort study that compared the oral mucosal changes that occurred with different tobacco products, we found that oral mucosal changes were seen in snus users, mostly male, who used at least 5–10 tobacco units (sachets) per day and had done so for least 5–10 years. All of the observed lesions were white and localized in the places where sachets are placed. Clinical examination and the collection of medical history and tobacco history are the main factors that help to locate places where white lesions could be present. White lesions are also known as smokeless tobacco keratosis [11]. Early lesions have a white appearance with minimal mucosal thickening, as is shown in our research. As the lesions progress, the mucosa becomes more keratotic and thicker [20]. Chewing products that also contain tobacco, such as areca nut and betel leaf (also called “paan”), are popular in Central Asia [21]. They affect oral mucosa more aggressively, leaving a diffuse, more wrinkled, thickened and corrugated surface texture, which eventually becomes squamous cell carcinoma [22]. Smokeless tobacco use is associated with an increased risk of oral squamous cell carcinoma, especially in women [23].

It should be considered that this research included Europeans that use smokeless tobacco products such as Swedish snus and nicotine pouches, and the results could have been different if we compared these to other types of smokeless tobacco products, such as Sudanese Toombak, paan, snuff, etc., as the products undergo different preparation and fermentation processes, and they harbor diverse bacterial microbiota [24].

In our study, although the most popular tobacco product remained as cigarettes, it was observed that e-cigarettes were the third most popular tobacco product. Worldwide e-cigarette use is increasing, and the interest of younger people in this product is also increasing, which is alarming [25]. In the 2019, Global Youth tobacco survey in Latvia, 14.8% of adolescents (aged 13–15) used cigarettes, but 18% used e-cigarettes [26]. In the same study that took place in 2014, it was seen that conventional cigarettes were used by 16.8% of adolescents (aged 13–15), but e-cigarettes were used by 10%. There has been an increase in the use of smokeless tobacco among adolescents: in 2014, 3.1% of adolescents used smokeless tobacco, but in 2019, 5.3% did so [27]. We can predict that, in the coming years, the distribution of tobacco product use will change, and it is likely that e-cigarettes and smokeless tobacco products or nicotine pouches will be the most popular products used in society.

In a systematic review conducted by Chiamulera et al., it was concluded that salivary biomarker levels (IL-8, IL-6, TNF alpha and IL-1 beta) were higher in individuals with potentially malignant oral disorders such as leukoplakia compared to healthy individuals. These levels were also higher for confirmed oral cancer patients compared to healthy individuals [16].

In the present study, we found an association not only between elevated IL-6 levels and oral mucosal changes but also between increased IL-6 and tobacco use. Salivary IL-6, as a pro-inflammatory cytokine, activates Janus kinases (JAK) and signal transducers and activators of transcription (STATs), which stimulate mitogen-activated protein kinase (MAPK), which activates cell proliferation and angiogenesis and supports cancer development. IL-8 influences cell proliferation and angiogenesis, but TNF alpha is responsible for cell survival, apoptosis and proliferation, which, overall, are involved in oral cancer development [28]. In our present study, the snus group had the highest values between all of the groups in terms of IL-1, IL-6, IL-8, TNF alpha and LRG1 levels. These results also correlate with mucosal changes in the snus group.

Although our study results do not associate increased salivary IL-8 and TNF alpha levels with oral mucosal changes or the use of tobacco, other studies that have determined salivary IL-8, TNF alpha and IL-6 levels in histologically approved oral leukoplakia, submucous fibrosis and lichen planus patients revealed that these biomarker levels were significantly increased in these populations [29].

It has been seen in other studies that smokeless tobacco users have lower biomarker levels than cigarette users, but these results are still higher than non-tobacco users. These results are dependent on whether on smokeless tobacco is used alone, together with cigarettes, etc. [30].

Our study showed that the highest value of LRG-1 protein was in the snus group; in comparison, in their research regarding oral squamous cell carcinoma candidate biomarkers in saliva, Kawahara et al. showed that LRG-1 levels are associated with the risk of developing oral squamous cell carcinoma [31]. A new method of examining individuals for the development of oral squamous cell carcinoma is to investigate salivary metabolites. The current results are promising, but further research in this topic should be carried out [32].

Our work, however, has several limitations. For example, currently, it is hard to separate Swedish snus users and nicotine pouch users. Historically, most respondents previously used Swedish snus that was illegally sold in Latvia, but in 2019, when nicotine pouches first entered the market, they switched or started to use both products at the same time. Because nicotine pouches are not tobacco products, they are legally sold in Latvia, and therefore, they are more readily available for chewing tobacco consumers. Both products look alike, both are placed under the lips and their pharmacokinetics are similar [33]. Compared to Swedish snus, nicotine pouches contain lower levels of toxic compounds [34]. However, as nicotine pouches are a new product in the field and research regarding this product is limited, we are not aware of all the possible risks they pose to human health.

Furthermore, other limitations to our study were that some respondents used more than one tobacco product daily. Based on the questions in the questionnaire, respondents who used more than one product at the same time were assigned to the group corresponding to the tobacco product they used the most tobacco units per day for, for example, if a respondent used 20 cigarettes per day and 5 sachets of smokeless tobacco, then this respondent was assigned to in cigarette group. It should be taken into the account that there were not many of these respondents (*n* = 5), but they could, in a small sense, affect the results.

Although our questionnaire was prepared with very specific questions concerning diet, oral habits, tobacco consumption, systemic diseases and medications, the possibility that the questionnaire did not cover all kinds of variables that would interfere with the results cannot be ruled out.

## 5. Conclusions

It can be concluded that e-cigarettes were used more by women, but snus was used more by men. Mucosal changes were seen in snus users, who were mostly men that had used 5–10 tobacco units per day for 5–10 years. Increased IL-6 levels in saliva were detected for respondents that experienced mucosal changes. Mucosal changes were white, leathery and localized at the place where snus sachets were placed. Snus users had high IL-1, IL6, IL8, TNF alpha and LRG1 levels in saliva. Saliva, as an easily available biofluid, could be used as a first tool to detect potentially precancerous signs, but it should be noted that further studies about this topic should be carried out regarding the use of this method in clinical use. The LRG1 marker cannot be used as a prognostic marker.

## Figures and Tables

**Table 1:** Smoking status of participants by gender.

Group	All (*n* = 76)	Women (*n* = 38)	Men (*n* = 38)
Age	24.46	23.71	25.21
Snus (%)	15.8	16.7	83.3
Cigarettes (%)	25	47.4	52.6
E-cigarettes (%)	10.5	62.5	37.5
Control group (%)	48.7	59.5	40.5

**Table 2:** Association between mucosal changes and average age, sex, and tobacco product type used.

Research Group	Sex, n	Average Age, Years	Tobacco Product Type
Snus	Women *n* = 2 Men *n* = 10	25.08	Swedish snus, nicotine sachets
Cigarettes	Women *n* = 9 Men *n* = 10	24.74	cigarettes
E-cigarettes	Women *n* = 5 Men *n* = 3	23.00	e-cigarettes
Control	Women *n* = 22 Men *n* = 15	24.43	-
Total	Women *n* = 38 Men *n* = 38	24.46	-
Association with mucosal changes	Women *p* < 0.05 Men *p* > 0.05 (Phi 0.285) *	*p* > 0.05 **	*p* < 0.05 (0.417) ***

* Fisher’s exact test used. ** Mann–Whitney U test used. *** Cramer’s V coefficient used.

**Table 3:** The patterns of mucosal changes of respondents.

Tobacco Type Used	Tobacco Units per Day Used	Duration of Tobacco Product Used, Years	Characteristics of Mucosa	Location
Snus	5–10	2–5	White, leathery lesion (Figure 1)	Above tooth nr. 13 to tooth nr. 22
Snus	5–10	2–5	White round lesion (Figure 2)	Above tooth nr. 13, 12
Snus	5–10	2–5	White, grainy lesion (Figure 3)	Above tooth nr. 13/12 and 22/23
Snus	5–10	5–10	White lesion	Above left and write upper premolars
Snus	5–10	5–10	White, linear lesions (Figure 4)	Right upper vestibule
Snus	5–10	15	White leathery lesions	Above upper central incisors
Snus	10–20	5–10	White, localized lesions	Above left lateral incisor
Snus	5–10	2–5	White, round localized lesions	Above left and right upper canines
Snus	5–10	2–5	White lesion	Above upper left and right premolars

**Table 4:** Average results of inflammatory biomarkers and LRG1 per group.

Group	IL-1 (pg/mL)	IL-6 (pg/mL)	IL-8 (pg/mL)	TNF-Alpha (pg/mL)	LRG1 (mg/mL)
Snus	105.14 (69.50)	177.43 (50.34)	767.43 (254.98)	445.08 (122.71)	306.35 (293.78)
Cigarettes	49.69 (82.17)	59.00 (94.59)	355.52 (120.67)	66.59 (92.44)	300.76 (170.90)
E-cigarettes	78.71 (92.37)	107.39 (70.92)	445.09 (135.12)	66.60 (181.63)	285.78 (130.46)
Control	21.32 (26.53)	40.54 (78.34)	281.85 (249.13)	60.22 (69.59)	265.53 (90.98)

Descriptive statistics used. Mean values and standard deviation.

**Table 5:** Association between mucosal changes and oral biomarkers.

	IL-1 (pg/mL)	IL-6 (pg/mL)	IL-8 (pg/mL)	TNF-Alpha (pg/mL)	LRG1 (mg/mL)
Correlation with mucosal changes	0.071 *p* > 0.05	0.344 *p* < 0.05	0.164 *p* > 0.05	0.143 *p* > 0.05	−0.105 *p* > 0.05
Correlation with tobacco product	0.039 *p* > 0.05	0.260 *p* < 0.05	0.01 *p* > 0.05	−0.102 *p* > 0.05	−0.052 *p* > 0.05
Correlation with duration of tobacco consumption	0.102 *p* > 0.05	0.191 *p* > 0.05	0.081 *p* > 0.05	−0.029 *p* > 0.05	0 *p* > 0.05
Correlation with tobacco units used per day	0.024 *p* > 0.05	0.144 *p* > 0.05	0.017 *p* > 0.05	−0.095 *p* > 0.05	−0.084 *p* > 0.05

Spearman’s rho correlation used.

## Data Availability

Not applicable.

## Supplementary Materials

The following supporting information can be downloaded at: https://www.mdpi.com/article/10.3390/dj10080154/s1, Figure S1: Respondent questionnaire about health, diet and tobacco consumption.
