# Supplementary material for: The Correlation of Swedish Snus, Nicotine Pouches and Other Tobacco Products with Oral Mucosal Health and Salivary Biomarkers
Source: Dent J (Basel). 2022 Aug 17;10(8):154. doi: 10.3390/dj10080154 (PMC9406994; doi:10.3390/dj10080154)
Supplement: Supplementary file 1 [file dentistry-10-00154-s001.zip › dentistry-1772662-supplementary.pdf]

Figure S1: Research questionnaire about health, diet and tobacco consumption.

The
Correlation of Swedish Snus, Nicotine Pouches and Other Tobacco Products with Oral Mucosal Health and Salivary Biomarkers.

* Required

1. Your Email adress *

2. Your name

3. Please write your telephone number (to schedule your dental check up): *

4. Gender *

Mark only one oval.

- Female
- Male

5. Age *

6. Nationality *

____

7. Occupation *

Check all that apply.

- [ ] Unemployed
- [ ] Pupil (in school)
- [ ] Student (in university)
- [ ] Athlete
- [ ] I work for a living
- [ ] Pensioner
- [ ] Other

8. What toothpaste do you use? *

____

9. How often do you brush your teeth? *

Mark only one oval.

- ( ) I don't brush at all
- ( ) Nonregulary
- ( ) Once a day
- ( ) Twice a day
- ( ) 3 times per day
- ( ) Other

10. Do you floss your teeth? *

Mark only one oval.

- ( ) Yes
- ( ) No

11. Do you use mouthwashes? *

Mark only one oval.

- Yes
- No

12. Do you use interdental brushes? *

Mark only one oval.

- Yes
- No

13. Do you use tongue cleaning brushes? *

Mark only one oval.

- Yes
- No

14. When was the last time you visited a dentist? *

Mark only one oval.

- I don't even remember
- In the last 6 months
- During the last year
- 2-3 years ago
- More than 4 years ago
- I have never visited a dentist

15. When was the last time you visited a hygienist? *

Mark only one oval.

- I don't even remeber
- In the last 6 months
- During the last year
- 2-3 years ago
- More than 4 years ago
- I have never visited a hygienist

16. Do you eat regularly on a daily basis? *

Mark only one oval.

- Yes
- No

17. Do you take snacks between meals? *

Mark only one oval.

- Yes
- No

18. Do you drink sweetened drinks every day (lemonades, juices, water with sugar, tea with sugar, coffee with sugar, etc.)? *

Mark only one oval.

- Yes
- No

19. How much sweetened drink do you drink every day? *

Mark only one oval.

- Up to 500 ml
- 500-1000 ml
- More than 1l
- I don't drink sweetened drinks

20. How much water do you drink every day? *

Mark only one oval.

- Up to 500 ml
- 500-1000 ml
- 1000-1500 ml
- More than 1500 ml
- I don't drink water daily

21. Do you drink alcohol on a daily basis? *

Mark only one oval.

- Yes
- No

22. Do you use chewing gum every day? *

Mark only one oval.

- Yes
- No

23. Do you have any dietary restrictions? If so, please explain

24. Are you a vegetarian or vegan? *

Mark only one oval.

- Yes
- No

25. Do you have diabetes? *

Mark only one oval.

- Yes
- No

26. Do you have asthma (or have you ever had asthma)? *

Mark only one oval.

- Yes
- No

27. Do you have reflux / GERD or stomach ailments? *

Mark only one oval.

- Yes
- No

28. Do you have heart disease / altered blood pressure? *

Mark only one oval.

- Yes
- No

29. Are you taking medication for heart health? *

Mark only one oval.

- Yes
- No
- No need

30. Do you have allergies? If yes, please list

____

31. Do you take antidepressants on a daily basis? *

Mark only one oval.

- Yes
- No

32. Are you currently taking intravenous drugs (cocaine, amefamamine, etc.)? *

Mark only one oval.

- Yes
- No

33. Do you smoke marijuana every day? *

Mark only one oval.

- Yes
- No

34. Have you used antibiotics in the last 3 months? *

Mark only one oval.

- Yes
- No

35. Are you pregnant right now? *

Mark only one oval.

- Yes
- No

Questions about tobacco

36. Do you use tobacco products on a daily basis? *

Mark only one oval.

- Yes
- No
- Occasionally at a parties, events

37. Have you used a tobacco or nicotine-containing product in your lifetime? *

Mark only one oval.

- Yes
- No

38. What tobacco products and products do you use every day: *

Check all that apply.

- [ ] Cigarettes
- [ ] Cigars
- [ ] Electronic cigarettes
- [ ] Snus
- [ ] Nicotine bags
- [ ] Hookahs
- [ ] Tobacco heating appliances
- [ ] Other
- [ ] I do not use tobacco products

39. How many cigarettes do you smoke a day? *

Mark only one oval.

- ( ) Up to 10 cigarettes per day
- ( ) 10-20 cigarettes a day
- ( ) More than 20 cigarettes a day
- ( ) I don't smoke

40. How many bags of snus or nicotine do you use per day? *

Mark only one oval.

- ( ) Up to 5 bags per day
- ( ) 5-10 bags per day
- ( ) More than 10 bags a day
- ( ) I don't use

41. Do you tend to reuse a bag of snus or nicotine? *

Mark only one oval.

- Yes
- No
- I don't use

42. Do you tend to use several bags of snus or nicotine at the same time? *

Mark only one oval.

- Yes
- No
- I don't use them

43. Do you use snus or nicotine bags at night? *

Mark only one oval.

- Yes
- No
- I don't use them at all

44. Do you use snus or nicotine bags during sports / training? *

Mark only one oval.

- Yes
- No
- I don't use them at all

45. How long have you been using tobacco products? *

Mark only one oval.

- About 1 year
- 2-5 years
- 5-10 years
- More than 10 years
- I don't use

46. The name of the tobacco product of your choice that you use most often:

____

47. Where do you buy tobacco products? *

Check all that apply.

- [ ] Sales outlets in Latvia
- [ ] Buy from acquaintances, friends
- [ ] I import from abroad myself
- [ ] I do not use tobacco products

48. How much does an average package cost?

____

49. Do you use chewing gum after using tobacco? *

Mark only one oval.

- Yes
- No
- I don't use tobacco

50. Do you brush your teeth after using tobacco? *

Mark only one oval.

- Yes
- No
- I don't use tobacco

51. Where you store your tobacco product: *

Check all that apply.

- Most often in the pocket
- In your handbag
- On the table, in the cupboard (at room temperature)
- In the refrigerator
- Elsewhere
- I don't use tobacco

52. Have you ever tried to quit using this product? *

Mark only one oval.

- Yes
- No
- I don't use tobacco

This content is neither created nor endorsed by Google.

Google Forms
